# Video livestreaming from medical emergency callers’ smartphones to emergency medical dispatch centres: a scoping review of current uses, opportunities, and challenges

**DOI:** 10.1186/s12873-024-01015-9

**Published:** 2024-06-11

**Authors:** Carin Magnusson, Lucie Ollis, Scott Munro, Jill Maben, Anthony Coe, Oliver Fitzgerald, Cath Taylor

**Affiliations:** 1 School of Health Sciences, University of Surrey, Guildford, Surrey UK; 2 South East Coast Ambulance Service NHS Foundation Trust, Crawley, West Sussex UK

**Keywords:** Emergency medical dispatch, Emergency medical services, Video livestreaming, Callers, Smartphone, Review

## Abstract

**Background:**

Timely dispatch of appropriate emergency medical services (EMS) resources to the scene of medical incidents, and/or provision of treatment at the scene by bystanders and medical emergency lay callers (referred to as ‘callers’ in this review) can improve patient outcomes. Currently, in dispatch systems worldwide, prioritisation of dispatch relies mostly on verbal telephone information from callers, but advances in mobile phone technology provide means for sharing video footage. This scoping review aimed to map and identify current uses, opportunities, and challenges for using video livestreaming from callers’ smartphones to emergency medical dispatch centres.

**Methods:**

A scoping review of relevant published literature between 2007 and 2023 in the English language, searched within MEDLINE; CINAHL and PsycINFO, was descriptively synthesised, adhering to the PRISMA extension for scoping reviews.

**Results:**

Twenty-four articles remained from the initial search of 1,565 articles. Most studies were simulation-based and focused on emergency medical dispatchers’ (referred to as ‘dispatcher/s’ in this review) assisted video cardiopulmonary resuscitation (CPR), predominantly concerned with measuring how video impacts CPR performance. Nine studies were based on real-life practice. Few studies specifically explored experiences of dispatchers or callers. Only three articles explored the impact that using video had on the dispatch of resources. Opportunities offered by video livestreaming included it being: perceived to be useful; easy to use; reassuring for both dispatchers and callers; and informing dispatcher decision-making. Challenges included the potential emotional impact for dispatchers and callers. There were also concerns about potential misuse of video, although there was no evidence that this was occurring. Evidence suggests a need for appropriate training of dispatchers and video-specific dispatch protocols.

**Conclusion:**

Research is sparse in the context of video livestreaming. Few studies have focussed on the use of video livestreaming outside CPR provision, such as for trauma incidents, which are by their nature time-critical where visual information may offer significant benefit. Further investigation into acceptability and experience of the use of video livestreaming is warranted, to understand the potential psychological impact on dispatchers and callers.

**Supplementary Information:**

The online version contains supplementary material available at 10.1186/s12873-024-01015-9.

## Background

In recent years, the rapid uptake in ownership of smartphones (with integral video technology) has influenced communication in healthcare. Many healthcare providers worldwide now offer remote patient consultations via video, in particular following the COVID-19 pandemic [1].

In the emergency medical dispatch context, timely and effective dispatch of appropriate emergency medical services (EMS) resources is critical, in order to save lives [2]. However, emergency medical dispatch response has been described as the weakest link in the EMS response chain [3]. There are several different dispatch systems around the world, commonly categorised into two different systems; the criteria-based dispatch (CBD) system, and the Medical Priority Dispatch system (MPDS) [4]. Both systems rely almost exclusively on audio and verbal information from the incidents, to determine the severity of the incident and inform dispatch decision making. Evidence suggests that dispatch and resource allocation is often inappropriate (either under or over resourced) resulting in additional costs and unfavourable clinical outcomes [5]. Similarly, support and instructions to bystanders and medical emergency callers (referred to as ‘callers’ in this review) to provide help at the scene also relies predominantly on verbal (usually telephone) communication. In recent years, the uptake of smartphone ownership means that visual as well as verbal information could be used at the scene. Video livestreaming (also referred to as ‘video’ in this review) from callers’ smartphones has the potential to facilitate the timely dispatch of appropriate EMS resources and improve treatment/care delivered by bystanders and callers, and to potentially assist callers, for example when carrying out CPR [6].

Previous reviews of use of video livestreaming have focussed exclusively on its use for out-of-hospital cardiac arrest (OHCA), in particular the use of video-instructed dispatcher-assisted bystander cardiopulmonary resuscitation (CPR) (V-DACPR). These reviews have reported that dispatcher video-instructed CPR significantly improved the chest compression rate compared to the audio-instructed method [7] and that V-DACPR significantly increased the return of spontaneous circulation (ROSC) and survival to hospital discharge [8]. A further review [9] examined the scope and nature of research publications on the topic of video emergency calls but did not synthesise or thematically analyse the findings in a way that could inform practice. None of the aforementioned reviews have examined the experiences of patients, healthcare staff or callers use of video livestreaming from medical incidents. This review therefore aimed to address these deficiencies by focusing on the current uses, opportunities, and challenges for using video livestreaming from callers’ smartphones to emergency medical dispatch centres (EMDCs).

## Methods

### Review question

The aim of this review was to map and synthesise the published international literature regarding video livestreaming (streaming of video in real time) between callers’ smartphones and EMDCs. The review questions were:

1. What are the current uses for video livestreaming from callers’ smartphones to emergency medical dispatch centres?
2. What are the challenges and opportunities when using video livestreaming between emergency medical dispatch centres and callers’ smartphones?

### Design

Arksey and O’Malley’s [10] five-stage scoping review method was used to guide the review. A scoping review synthesises and maps the literature in a particular area of practice, highlights the different types of evidence and identifies knowledge gaps in an emerging field. We chose to do a scoping review, as there had been limited research concerning video livestreaming in the dispatch context. There was a need to get an initial understanding of the topic area to understand the scope and nature of current evidence. We adhered to the PRISMA extension for scoping reviews [11]. The PRISMA-ScR checklist can be found in Supplementary File 1. The review protocol has not been published.

### Stage 1: identifying the research question

We started by identifying the research question following initial scoping of the evidence. Due to limited number of relevant articles, it was agreed to include all types of clinical focus (such as trauma or CPR), as well as both real-life and simulation-based studies. We also agreed to focus on medical emergency lay callers only as this was our primary interest.

### Study types and characteristics

This review included quantitative, qualitative, and mixed methods primary research studies, published in peer-review journals in any country in the English language between 2007 (introduction of smartphone video technology) and 2023. Reviews were excluded. Both ‘real-life’ and simulation studies were included due to the limited evidence base available for real-life studies only, and also due to the opportunity for learning from simulation settings, but papers were synthesised, and results are presented separately.

### Stage 2: identifying relevant studies

MEDLINE; CINAHL and PsycINFO databases were searched in February 2023. Key words in context to the title/abstract were identified. Key terms included emergency medical dispatch, emergency medical services, air ambulance, cell phone, smartphone, telemedicine, video, livestreaming, and relevant Medical Subject Headings (MESH) using Boolean operators. An example Medline string can be found in Supplementary file 2.

### Stage 3: study selection/screening

**Fig. 1:**
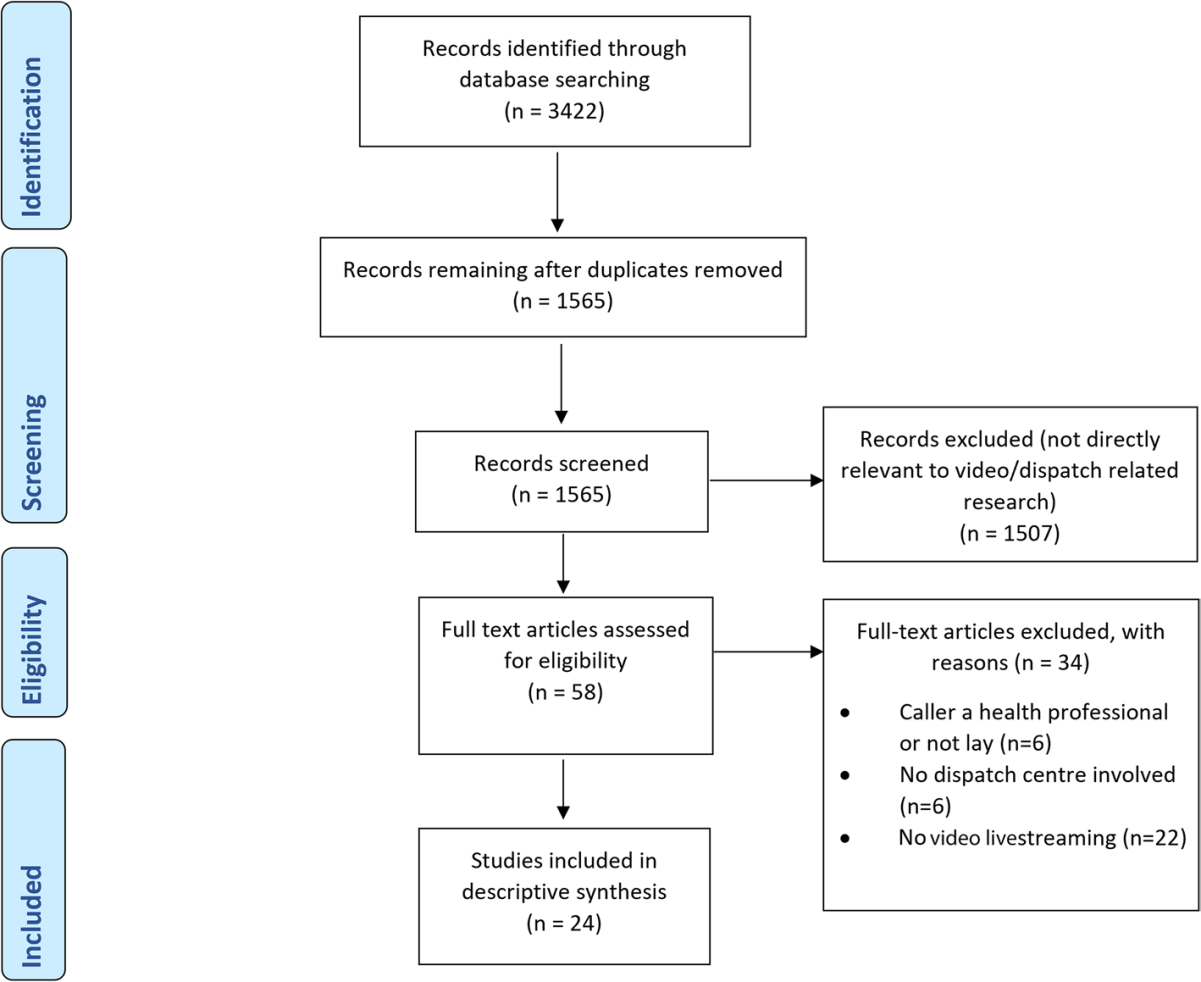
PRISMA flow diagram^19^

Once the search was completed, citations were uploaded to Zotero, the reference management software and duplicates were removed resulting in 1565 papers. Title and abstracts were screened, and articles removed if they were not directly relevant to video livestreaming or dispatch. This resulted in 58 remaining articles. There was much literature covering specific video-diagnostic equipment such as laryngoscopy and other video devices used in the pre-hospital and emergency care environment, that was not relevant to this study. These were removed. Full-text review of the remaining articles (*n* = 58) were independently assessed (CM & LO) for eligibility using the inclusion/exclusion criteria. There was 100% agreement regarding inclusion/exclusion of these articles. In total, 24 studies were included (see Fig. 1 Prisma flowchart).

### Review inclusion & exclusion criteria

Inclusion criteria included:

1. Studies concerning use of video livestreaming between a lay caller and an emergency medical dispatch centre (or participant acting in the role of emergency medical dispatcher/call taker in simulation studies) AND
2. Studies where the caller was a lay person (or acting in the role of a lay person in simulation studies) AND
3. Studies set in an emergency medical dispatch centre or simulating that setting.

Exclusion criteria included: caller being a health professional or not a lay caller (such as the police); research not involving emergency dispatch or an emergency medical dispatch centre; or did not involve video livestreaming.

### Stage 4: charting the data/data extraction

Data were extracted independently by the first author (CM all articles) and repeated by one of two research paramedic co-authors (AC and OF, each randomly allocated 50% of articles) using a bespoke data extraction tables in Word (see Tables 1 and 2). These were designed to comprehensively capture generic study information (i.e., aims, objectives, study settings/country etc.), as well as key study characteristics such as design/methodology, and information about the study population. We also extracted information about whether the study was real-life or simulation, whether the video communication was one- or two-way, study clinical focus, and reported key challenges and opportunities to using video livestreaming. Data extraction was compared between the three reviewers and any differences were discussed and agreed between reviewers. We did not appraise the methodological quality of the articles, consistent with scoping review guidance [10].

**Table 1:** Real-Life Studies - Characteristics of sources of evidence and results

Author, Year	Origin/ Country	Aim/Purpose	Methodology/ Study Design	Sample Size and Study population	Uses one- or two-way video	*Results* Uses/ clinical focus	*Results* Challenges and Opportunities
1. Bell et al. 2021 [12]	England	Investigating patient and staff acceptability of video triage following 999 calls to medical emergency dispatch centre, and the safety of the decision-making process.	Quantitative, Service Evaluation	Clinicians documented 1073 triage calls. Survey (*N* = 40/201, response rate 19.9%) with callers/patients	Not reported	All type of cases	*Challenges*: Some callers reported technical issues, such as video freezing. *Opportunities*: Clinical staff reported that video triage improved clinical assessment and decision making. Patients who received a video triage call and responded to the survey were satisfied with the video call technology (98%, *n* = 39/40). Over half of calls were categorised to higher priority after video consultation. Substantial number of calls initially categorised as category 5 (lowest priority so called “hear and treat”) were upgraded to category 2 (190/850, 22.4%) or 3 (273/850, 32.1%) (as self-reported by clinician, not externally validated).
2. Idland et al. 2022 [13]	Norway	Explore how the dispatchers experience using video streaming as an additional tool in medical emergency calls.	Qualitative	Interviews with 25 dispatchers (nurses and medical technicians)	One-way	All type of cases	*Challenges*: Some dispatchers believed that video streaming was a tool too new for them, feeling a need for more experience to be comfortable with the usage of video. *Opportunities*: Dispatchers experienced that video streaming improved decision-making regarding further care for the patient. Several of the dispatchers interviewed reported that they had experienced the caller to be more reassured and satisfied when using video.
3. Lee et al. 2021 [14]	South Korea	To assess whether video‑instructed CPR improved neurologic recovery and survival to discharge compared to audio CPR	Quantitative, retrospective cohort study	2109 cases/patients. 387 patients (18.3%) received video-instructed Dispatcher Assisted CPR	Not reported (article refers to ‘video call’)	CPR	Challenges: Public perception and number of smartphones with video-functionality. Smartphone rate in the population of Korea was approximately 95% at the time of the study. Opportunities: Favourable neurologic outcome was observed more in patients who received video-instructed DA-CPR (*n* = 75, 19.4%) than in patients who received audio-instructed DA-CPR (*n* = 117, 6.8%). The survival to discharge rate was also higher in video-instructed DA-CPR (*n* = 105, 27.1%) than in audio-instructed DA-CPR (*n* = 211, 12.3%).
4. Lee et al. 2020 [15]	South Korea	Comparing real-world effects of audio-instructed dispatcher-assisted cardiopulmonary resuscitation (DA-CPR) and video-instructed DA-CPR.	Quantitative, retrospective cohort study	Eligible OHCA patients (*n* = 1720)	Two-way	CPR	*Challenges*: Compared to audio-instructed DA-CPR, video-instructed DA-CPR was not associated with survival improvement in this observational study. There needs to be more than two people/callers present – one providing chest compressions and the other using mobile phone. *Opportunities*: video-instructed DA-CPR did not delay the time to first chest compression.
5. Linderoth et al. 2021 [6]	Denmark	Whether live video from bystanders’ smartphones changed emergency response and was beneficial for the dispatcher and caller.	Quantitative, cohort study	111 callers 637 questionnaires from dispatchers (dispatchers completed one questionnaire for each video call). All medical dispatchers (n not reported) in participating EMS could add live video to emergency call	One-way	All types of cases	*Challenges*: Using live video is challenging using existing dispatch protocols. Live video was only used in 1.4% of the calls. Feedback from dispatchers suggested they did not find video necessary in many calls. *Opportunities*: The dispatchers’ assessment of the patients’ condition changed in 51.1% of the calls (condition more critical in 12.9% and less critical in 38.2%), resulting in changed emergency response in 27.5% of the cases after receiving the video. 97.3% (*n* = 108/111) of callers felt live video should be implemented. Video was added more frequently by the dispatchers in cases with sick children or unconscious patients.
6. Linderoth et al. 2021 [16]	Denmark	If live video streaming from the bystander’s smartphone to a medical dispatcher can improve the quality of bystander CPR.	Quantitative, retrospective cohort study	52 Video recordings. Dispatchers (specially trained registered nurses and paramedics), 90 bystanders	One-way	CPR	*Challenges*: Although not measured, the researchers noted that in some situations, the bystander did not follow dispatcher instructions, for example “press harder” during CPR. *Opportunities*: Once video instruction started correct or improved CPR was shown in 81.1% for hand position, 77.7% for compression rate, and 58.9% for compression depth. Live video streaming scenes of cardiac arrest to medical dispatchers is feasible.
7. Neustaedter et al. 2018 [17]	Canada	Understand call taker and dispatch work practices, the potential benefits of video calling for 911, and the challenges that 911 calls might create.	Qualitative	18 Call takers/Dispatchers	N/A. Study observed normal practice (no video) and interviewed dispatchers regarding potential use of video for dispatch.	All type of cases	*Challenges*: Video calls have the potential to introduce issues around call taker stress, control, information overload and privacy. *Opportunities*: Video calls could provide contextual information about a situation and help to overcome call taker challenges with information ambiguity.
8. Steen-Tveit et al. 2021 [18]	Norway	An analysis of work practices in command-and-control centres (CCC) in Norway and documenting experiences from early-stage adoption and use of a live video system.	Qualitative	Interviews (n not stated) with Emergency dispatchers. Observations in command-and-control centres	One-way	All type of cases	*Challenges*: Live video sometimes caused emotional stress for dispatchers, leaving visual impressions, they were not prepared for. *Opportunities*: The respondents reported how video supported situational awareness: (1) to assess if this is a situation to respond to at all; (2) to evaluate the degree of different situational elements (e.g., the severity of bleeding); and (3) get an overview of the context and adapt measures and advice according to what they see.
9. Ter Avest et al. 2019 [19]	England	Acceptability and feasibility to of using live video footage from the mobile phone of an emergency caller as a HEMS dispatch aid.	Service Evaluation Quantitative, Prospective	Dispatchers (Helicopter Emergency Medical Service (HEMS)) 21 Emergency calls	One-way	All type of cases	*Challenges*: In two calls (*n* = 2/19) there was no data coverage, precluding video streaming from scene. *Opportunities*: Video footage from scene was successful in 19/21 calls. Video was readily accepted by the caller. No caller refused to transmit live video. All callers understood instructions. Dispatcher found it helpful to visualise patient and scene.

**Table 2:** Simulation Studies - Characteristics of sources of evidence and results

Author, Year	Origin/ Country	Aim/Purpose	Methodology/ Study Design	Sample Size and Study population	Uses one- or two-way video	*Results* Uses/ clinical focus	*Results* Challenges and Opportunities
10. Bang et al. 2020 [20]	Korea	Investigate the feasibility of mobile videocall guidance to facilitate Automated External Defibrillator (AED) use by laypersons.	Mixed methods, randomised, prospective	90 Laypersons (adult college students acting as laypersons) Dispatchers (researchers)	Not reported	AED, cardiac arrest	*Challenges*: Time interval to defibrillation was significantly longer in the mobile video call-guided group. *Opportunities*: Performance scores in the checklist for using AED were higher in the mobile video call-guided group, especially in the category of “Power on AED” and “Correctly attaches pads” than in the other groups.
11. Bolle et al. 2011 [21]	Norway	Whether video calls from mobile phones could improve the confidence of lay rescuers.	Quantitative	Survey with 180 school students as lay bystander. 6 Nurse dispatchers (1–10 years experience)	Two-way	CPR	*Challenges*: Some rescuers/callers felt video was disruptive, as moved attention away from simulated incident. *Opportunities*: Video improved self-reported confidence of rescuers. Rescuers in both groups believed video calls were superior to audio calls during medical emergencies. Most participants found instructions easy to use.
12. Bolle et al. 2009 [22]	Norway	Study the quality of simulated dispatchers assisted CPR when guidance was delivered to rescuers by video calls or audio calls from mobile phones.	Randomised Control Trial (RCT), mixed methods	180 school students as lay bystander. 6 Nurse dispatchers (1–10 years’ experience, 60 cardiac arrest simulations	Two-way	CPR	*Challenges*: The mobile video telephones and the network bandwidth used during this study did not allow very high-quality picture. *Opportunities*: Video groups had a significantly shorter CPR ‘hands-off time’. Dispatchers adapted their instructions based on the feedback they receive from the rescuers.
13. Ecker et al. 2022 [23]	Germany	To evaluate the dispatchers’ attitudes toward Video CPR (V-CPR)	Quantitative	50 volunteers acted as bystanders. 4 dispatchers (average 10 years dispatching experience and trained paramedics) 49 scenarios	One-way	CPR	*Challenges*: In 30% of the scenarios the dispatchers agreed that they found the video-assisted method more stressful/exhausting. Dispatchers also felt that video streaming could interfere with normal protocol. *Opportunities*: In 80% of the scenarios dispatchers strongly agreed that V-CPR was helpful and that their feedback to the lay bystanders improved CPR quality. In 51% of the scenario’s dispatchers agreed that video streaming helped with diagnosis.
14. Ecker et al. 2021 [24]	Germany	Whether the dispatcher recognized correct and incorrect resuscitation performance.	Quantitative, Randomised	2 dispatchers. (study team acting as bystander, 54 study sites attempting video telephony. 46 successful video calls	One-way	CPR	*Challenges*: In 8 attempts of calls (14.8%) software failures of app or server system problems did not facilitate video connectivity. *Opportunities*: Dispatchers identified correct compression frequency in 87.5% of the cases. Audio quality was rated as excellent in 98.1% of the calls.
15. Igarashi et al. 2022 [25]	Japan	Whether video calls with dispatchers improve the quality of first aid for infants with foreign body airway obstruction (FBAO).	Quantitative, (RCT)	70 callers (acted by students) 4 dispatchers (acted by faculty members) 17 simulations in the video-call groups and 16 in the voice-call groups	Not reported	Foreign Body Airway Obstruction (FBAO) (infants)	*Challenges*: Video-calls were unsuccessful in 24% of the calls, as participants did not pick up the call. *Opportunities*: Oral instruction communicated by video calls improved the quality of first aid for infants with FBAO.
16. Johnsen and Bolle, 2008 [26]	Norway	How mobile phone video-calls compares with traditional phone calls for DACPR.	Qualitative	6 dispatchers (following simulated cardiac arrest)	Two-way	CPR	*Challenges*: Loss of the dispatcher’s identity protection, by being visible to callers, was described as a disadvantage of video-calls. Video-calls introduced ‘‘a new point of focus’’, and at times the protocol was left in favour of watching the video. *Opportunities*: Video-calls are useful for obtaining information and provides adequate functionality to support CPR assistance. CPR assistance becomes easier. The CPR might be of better quality.
17. Kim et al. 2021 [27]	South Korea	Comparing CPR performance when dispatcher provides audio instructions only and when both audio and video instructions are given.	Randomised, Mixed methods	24 college students. CPR performance evaluation using manikin. AED delivered via drone.	Two-way	CPR	*Challenges*: Some first responders (students acting as) felt embarrassed by the unexpected situation, as it was unfamiliar. *Opportunities*: Video-based instruction more effective in the number of chest compressions, chest compression rate, and interruptions. The dispatcher’s instructions on how to properly perform CPR provided comfort to the rescuer that they were not alone.
18. Lee et al. 2021 [28]	South Korea	To develop new audio call-to-video call transition protocols and test its efficacy and safety.	Quantitative, RCT	131 volunteers as lay bystander. 2 dispatchers	Not reported (article refers to ‘video call’)	CPR	*Challenges*: The dispatch center in Seoul cannot directly change from audio to video call without discontinuing the phone call. This has the risk of losing connection between the dispatcher and bystander. *Opportunities*: Participants in the V-DACPR groups performed higher quality chest compressions with higher appropriate hand positioning and deeper compression depth.
19. Melbye et al. 2014 [29]	Norway	If video can be used between lay bystanders and Emergency Medical Dispatcher’s (EMDs) for initial emergency calls under suboptimal sound and light conditions.	Quantitative	90 bystanders (volunteers acting as) 2 dispatchers (acted by medical students)	Two-way	Foreign body in mouth	*Challenges*: Night-time group had lower video quality. More repetitions of instructions needed when there was a lot of background noise. *Opportunities*: 97% of calls perceived by EMDs as very or fairly easy to understand.
20. Perry et al. 2020 [30]	Israel	To compare CPR effectiveness under three conditions: telephone-instructed, video-instructed, and video-instructed with the filming protocol.	Quantitative	Dispatchers (experienced Emergency Medical Technicians acting as) Bystanders (played by students). 49 scenarios	One-way	CPR	*Challenges*: The depth and rate of compressions did not improve in the filming protocol (developed to help video communication) condition. *Opportunities*: Compared with telephone-instructed CPR, the filming protocol (developed to help video communication) improved the proportion of time in which the bystander’s hands were in the correct position during chest compressions.
21. Peters et al. 2022 [31]	Belgium	Evaluate the impact of adding video conferencing to dispatcher-assisted telephone CPR on pediatric bystander CPR quality, in trained and untrained volunteers.	Quantitative, Prospective, randomised	8 dispatchers (trained in study protocol) 120 subjects	One-way	CPR (paediatric)	*Challenges*: Subjects in the video groups had a lower fraction of minute to ventilate as compared with the telephone groups. *Opportunities*: In dispatcher-instructed children CPR simulation, using video assistance improves cardiac arrest recognition and CPR quality with more appropriate chest compression technique and ventilation delivery.
22. Stipulante et al. 2016 [32]	Belgium	To validate an original protocol of Video-CPR and to evaluate the potential benefit in comparison with classical telephone-CPR.	Quantitative, Prospective RCT	120 rescuers (Students acting as) 5 dispatchers (trained to follow 2 different algorithms)	Not reported (reports ‘video -conferencing’)	CPR	*Challenges*: Video did not offer advantage in checking for responsiveness compared to telephone CPR. *Opportunities*: The video CPR protocol allows bystanders to reach compression rates and depths close to guidelines and to reduce ‘hands-off’ events during CPR. The mean chest compression rate was higher in the v-CPR group.
23. Yang et al. 2008 [33]	Taiwan	Assess the impact of adding interactive video communication to dispatch instructions on the quality of rescue breathing in simulated cardiac arrests.	Quantitative, RCT	96 rescuers (volunteers acting as). The role of the dispatcher was played by an emergency physician familiar with the DA-CPR protocol	Two-way (not reported, but state that “dispatcher demonstrated two initial rescue breaths via video” indicating two-way)	Rescue breathing, cardiac arrest	*Challenges*: The video group spent longer time to open the airway (59 s vs. 56 s, *P* < 0.05) and to give the first rescue breathing (139 s vs. 102 s, *P* < 0.01). *Opportunities*: More people in the video group opened the airway properly than those in the voice group (95.3% vs. 58.5%, *P* < 0.01) and a tendency towards better nose-pinch (97.7% vs. 86.8%, *P* = 0.06).
24. You et al. 2008 [34]	South Korea	To evaluate whether using an AED with video telephony-directed cellular phone instructions for untrained laypersons would increase the chances of successful use of AEDs.	Quantitative, Prospective	52 callers (volunteers) 1 dispatcher	Two-way (not reported, but state that “dispatcher gave proper command via the video telephone”, referred to as correction by video telephony).	Automated external defibrillator, cardiac arrest	*Challenges*: The results indicated that video telephone-directed use of AEDs requires additional time. The mean (SD) time to the first defibrillation was 131.8 (20.6) s (range 101–202) which was longer than the results of previous studies. *Opportunities: 3*7 participants (71.1%) were corrected by video instructions for correct pad placement.

### Stage 5: collating, summarising and reporting the results

The findings were synthesised descriptively (real-life and simulation separately) according to Arksey and O’Malley’s [10] framework using thematic analysis to answer the review questions. Key themes were identified (and sub-themes). Results were organised and mapped according to the review questions (opportunities and challenges) for each theme. This process was iterative and involved updating data extraction tables and additional re-reading of the papers to make sure that all relevant data relating to the themes or main themes had been included in the analysis.

## Results

**Fig. 2:**
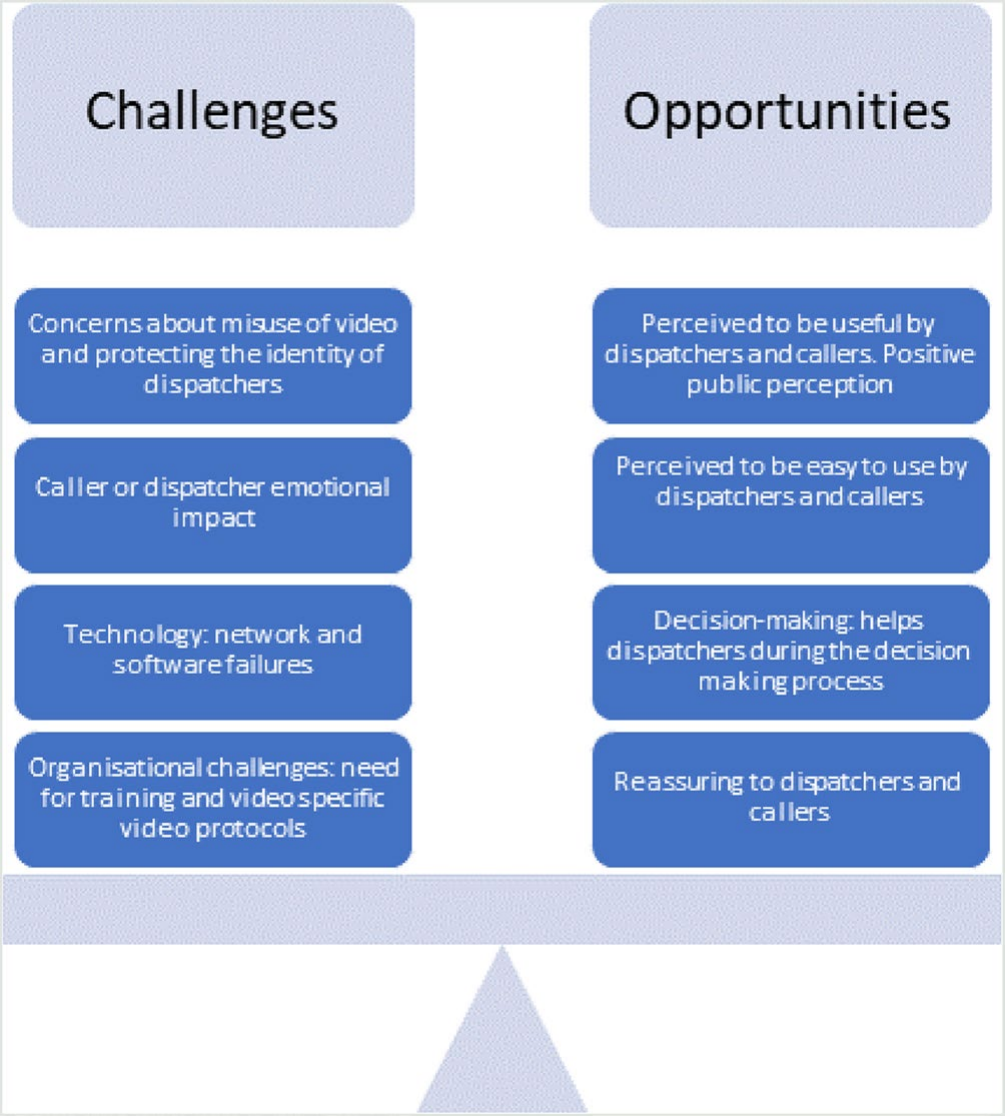
Challenges and opportunities to using video livestreaming

In total, 24 studies were included (see Fig. 1, PRISMA Diagram). Nine of the studies were based on real-life clinical practice (see Table 1) and fifteen were simulation studies (see Table 2). Ten of the fifteen simulation studies used comparison groups (nine used audio/voice calls and one study suboptimal light and sound conditions) as part of the research design. Only two of the real-life studies [14, 15] used comparison groups, both focussing on dispatcher-assisted CPR clinical outcomes. In total, half of the included 24 studies used comparison groups and the other half relied on less robust study designs and used self-report data only. Most studies were conducted in Norway (*n* = 6) and South Korea (*n* = 6), followed by Belgium (*n* = 2), Denmark (*n* = 2), England (*n* = 2), Germany (*n* = 2). One study was conducted in each of the following countries: Canada [17], Israel [30], Japan [25] and Taiwan [33]. Nine of the studies reported using one-way video (caller not able to see the dispatcher), seven studies used two-way video communication (caller and dispatcher could see each other) and the remainder did not report mode of use (see Tables 1 and 2). Most included studies were quantitative (*n* = 17), four were qualitative, and three were mixed methods. The design, source characteristics and results are summarised in Table 1 (real-life studies) and Table 2 (simulation studies). The results of the thematic synthesis of findings are organised under four main heading, and key findings are presented in Fig. 2 (below).

### Current uses of video livestreaming between callers and emergency medical dispatch centres

In the nine real-life studies, three focussed on CPR only [14–16] and six had a broad clinical focus (with limited further detail included in the papers) ranging from trauma incidents to lower priority calls (see Table 1). These studies were undertaken in various countries including Norway, where a study had piloted video in four medical emergency medical communication centres [13]; Denmark [6], where dispatchers were able to decide when to use video streaming, and reported that video was used more frequently in cases with sick children or unconscious patients; and Seoul, South Korea, video was added to their dispatcher-assisted CPR programme in 2017 [15].

In the 14 simulation studies, ten focused on dispatcher-assisted CPR (see Table 2), two on incidents simulating foreign bodies in mouth (used as simulation scenario) [25, 29], and two investigated the feasibility of using video call to guide lay people using automated external defibrillators (AEDs) following simulated cardiac arrests [20, 34]. The majority of simulation studies focussed on adult casualties, but two used paediatric cases [25, 31].

### Emergency medical dispatchers’ perspectives: opportunities and challenges

Over half of the papers (*n* = 14/24) included some information (though few had this as the study focus) on the views of the dispatchers (or those acting as dispatchers in simulation studies) about video livestreaming. Perspectives reported in these papers were overwhelmingly positive, with dispatchers reporting video livestreaming to be acceptable and easy to use, reporting various benefits of video livestreaming including its use as an aid for emergency medical dispatch decision-making, video dispatcher-assisted CPR improving caller technique when performing CPR, more accurate patient assessment and/or supporting better/more effective decision-making. Challenges included the potential emotional impact of seeing traumatic incidents that dispatchers would normally only ‘hear’, the potential for additional fatigue and workload, and concerns about the potential misuse of video were also reported. There was no evidence provided in any of the papers to support these concerns other than perceived potential impact. However in one real-life Norwegian study [18] dispatchers referred to colleagues having visual impressions that they were not prepared for.

#### Real-life studies

Clinicians reported that video ‘consultations’ (following medical emergency calls) were superior to audio only [12], easy to use [26], and that they wanted to use video in the future, especially for dispatcher-assisted CPR [23]. Dispatchers reported finding video livestreaming extremely useful [6], that it improved communication with callers [13], and also provided dispatchers with general reassurance in their decision-making [13]. Additional benefits included the ability to make a more thorough assessment of patients’ needs compared to telephone alone [12, 13]. Dispatchers also reported that video streaming helped them assess the quality of CPR, obstructed airways, or position of the patient [16], and more easily evaluate different situational elements (such as bleeding or context of incidents) [18]. There was some preliminary evidence that video livestreaming impacted positively on dispatch decisions, prioritisation, and/or resource allocation. For example, in one study the medical dispatchers changed the dispatch response in approximately one quarter of calls after viewing the video [18]. In another study, dispatchers reported that video supported more precise resource allocation [13]. *Challenges* reported in real-life studies (self-report) included the potential emotional impact of viewing video footage [18], in particular when the call involved children [13]. In this Norwegian study, dispatchers could choose to use live video as an additional tool for communication, and a few of the dispatchers interviewed described that they felt uncomfortable activating video when children were involved [13]. Additionally, some dispatchers reported in interviews that using video livestreaming was more time-consuming and might divert their attention, potentially leading them to forget other tasks [13], increased their workload [6] and created potential information overload, although the dispatchers in the study [17] had never used video streaming.

#### Simulation studies

In simulation studies, video was reported to be superior to audio alone, helped to clarify misunderstandings with callers and motivated callers to continue with CPR [22–24]. Dispatchers reported that video helped them to save time gathering information from the callers, as they did not need to ask all of the standard questions [26]. Half of the dispatchers in a German simulation study (51%) agreed that video “supported them in making a diagnosis” [24]. Only two of the simulation studies covered challenges with video streaming from the dispatchers’ perspective [23, 26]. Ecker et al. [23] surveyed dispatchers following simulated cardiac arrests and 30% agreed that they found using live video more stressful or exhausting compared to normal telephone (audio) protocol (simulation used video only, we make the assumption that the dispatchers compared this to their normal audio practice). Similarly, in Johnsen and Bolle’s [26] study, some of the dispatchers interviewed felt that video had the potential to cause extra strain and stress, for example, when witnessing deaths. The dispatchers in this simulation study were also worried about the videos potentially being misused and distributed by callers to mass media, as well as the loss of identity protection, as in this study dispatchers were visible to callers [26].

### Caller perspectives: opportunities and challenges

Ten studies included perspectives from or regarding the callers (or those acting as callers in simulation studies). Most of these studies (*n* = 6) reported proxy views of the callers’ perspectives from dispatchers, for example describing their experience of dealing with the caller, and sometimes speculating what callers may do if they added video to a call. The evidence in these papers suggested that callers in both real-life and simulation studies had positive attitudes and experiences of video livestreaming. On the basis of these studies, video streaming technology appears largely acceptable to callers, and few challenges were reported. Callers were generally positive, reported feeling reassured by the video, comforted by not being alone, and satisfied with the help they received. Very few challenges from the caller perspective were reported in the included articles, but some contained instances when video was reported to potentially increase callers’ stress, was disruptive or divided/diverted attention from the scene.

#### Real-life studies

Four of the real-life studies included assessment of acceptability to callers or satisfaction with the use of video livestreaming (all these studies used self-report, see further below). Callers found video reassuring, comforting and not feeling “alone” [13], and enabled them to express their needs in a better way [12]. Linderoth et al. [6], surveyed callers in Denmark following real-life emergencies using live video, and nearly all (97.3%) reported that live video should be implemented. Although Ter Avest et al. [19] did not specifically ask callers about their experience, all of the callers in the study (*n* = 21) were willing to attempt video livestreaming and the dispatchers reported that all callers were able to follow their instructions. Dispatchers rated their perception of callers’ acceptability of using video as “excellent” in all calls [19]. An interesting observation was made in a real-life study [14] in Seoul, South Korea, where video-instructed dispatcher-assisted CPR has been well established in practice for some time. It was noted anecdotally that increasing public awareness of its use through national news items reduced the tendency of callers to reject the change from audio to video instructions, hence improving acceptability. *Challenges* reported in real-life studies included dispatchers perceiving that, at times, the use of video increased callers’ stress [13] and some callers reporting technical difficulties such as video freezing [12].

#### Simulation studies

As per the real-life studies above, callers in simulation studies have reported that sharing the scene with the dispatchers through video gave them comfort that they were not alone [27] and feeling reassured that dispatchers “could see whether I did the right thing” [12]. Most of the callers in Bolle’s et al.’s [21] Norwegian study reported that using video was better than standard telephones during medical simulated emergencies and improved their confidence when performing CPR. *Challenges* reported in simulation studies were very rare, although one survey participant (caller) in a Norwegian simulation study reported that they were concentrating more on recording the video than the situation [21]. In a Korean study [27], callers reported being concerned about how they would manage the situation if connection was lost during a video call, though none of them reported that this had happened.

### Organisational perspectives: opportunities and challenges

Although none of the studies specifically focused on organisational issues, or systematically collected such data, ten studies offered important insights into the challenges and opportunities of using video livestreaming from an organisational perspective. The reported evidence in these papers suggest a need for training, video-specific protocols and a need to consider how best to implement video livestreaming into existing organisational structures. No specific organisational benefits were reported in the studies, although the benefits reported by callers and dispatchers earlier is suggestive of benefits to the organisation, for example by improving resource allocation.

#### Real-life studies

Linderoth et al. [6] reported that video livestreaming could potentially interfere with existing symptom-based dispatch protocols; it was observed by the researchers that some of the dispatchers appeared to avoid using video livestreaming during particularly busy times. Other studies have supported the need to implement dispatch protocols and guidelines for the specific purpose of video livestreaming, as well as highlighting the need to fully integrate video features into existing IT-systems and processes [18]. Dispatchers have highlighted the need for experience in using the new technology [13] and the authors in another study suggested that the helicopter emergency medical service (HEMS) dispatchers in their pilot study needed to be provided with adequate support when potentially witnessing images that were unusual in their role [19]. Although not specifically measured, the authors in one of the real-life studies noted that there had been positive effects between the dispatcher and caller via video-instructed CPR, such as providing real-time feedback [14].

#### Simulation studies

Similar findings about the importance of protocols and training were reported in the simulation studies. Ecker et al. [24] argued that training and additional support is needed to help dispatchers to develop new skills in using video. In another study, it was argued that training was also needed to help dispatchers integrate video CPR into existing workflows [23]. Stipulante et al. [32] developed and validated a protocol for video-assisted CPR and the new protocol supported improvement in some of the CPR performance indicators. Similarly, Perry et al. [30] used a ‘filming protocol’ for dispatcher video-assisted CPR and findings showed that the bespoke protocol improved CPR effectiveness. Perry et al. [30] also stressed that to maximise the use of the new technology, special attention is needed to implement video streaming in practice.

### Technological factors: opportunities and challenges

Ten studies (five real-life and five simulation studies) included findings regarding the streaming technology, such as impact of lighting conditions, visibility, and sound quality. The few technical challenges that were identified in this review included problems with SMS/links, the positioning of the video camera, and lack of network coverage, although this was rare. Despite some difficulties in relation to these issues, in the majority of uses within all studies (both real-life and simulation studies), few technical barriers were reported. Both real-life and simulation studies reported high video call success connection rates.

#### Real-life studies

Two studies reported video call success rates; nineteen out of 21 calls were successful in obtaining video from the scene in a small feasibility study [19] and live video succeeded in over 80% of calls (*N* = 838) in a study in Denmark [6]. The small number of unsuccessful calls in these two studies were reported to be due to technical issues, lack of caller skills or callers not receiving the SMS with the link to activate the video. Some dispatchers in a Canadian study were worried about potential technical challenges with the CAD (computer aided dispatch) system and transferring calls, although they had never personally used video for dispatch purposes [17].

#### Simulation studies

One study [29] specifically investigated using video livestreaming under different sound and light conditions. The authors concluded that video calls can be used successfully in suboptimal conditions, such as during night-time, poor lighting, and noisy conditions. Dispatchers in a simulation study carried out in Germany judged both the video audio quality and the video quality as excellent in nearly all calls. Several of the simulation studies explored video call success connection rates. Video calls succeeded in 76% of calls in a Japanese study [25] and in 85% of calls in a German study [24]. The technical reasons for unsuccessful calls were due to network failure, application software failures, and IT server problems. Dispatchers interviewed in a Norwegian study [26] reported some technical challenges, such as the image quality depending on how steadily the camera was held. Others reflected that if the call had been outside, the wind and noise from people and cars may be disruptive [26].

## Discussion

Our review has shown that despite the increased uptake of video livestreaming in emergency services, the evidence base remains sparse. Much of the previous research focuses on one aspect of emergency care, the use of CPR, and many of the studies are simulation-based rather than real-life clinical settings. Furthermore, methodological limitations have made attribution of findings to the use of video challenging, such as the lack of control groups or use of randomised designs, and there is a reliance on self-reported data.

Nevertheless, with these caveats, evidence from this synthesis suggests that video streaming is acceptable and easy to use to both callers and dispatchers and could be beneficial in relation to supporting video dispatcher-assisted CPR, and reassuring dispatchers and callers. There is limited research regarding callers overall experiences of calling EMDCs, but a literature review [35] found that bystanders at motor vehicle accidents often experienced fear of liability or fear of further harming the victim/patient. Video livestreaming offering reassurance to callers may provide additional benefits in these situations. Our results highlighted that callers were generally positive to video livestreaming, but most of the evidence relied on proxy views of the callers’ perspectives from dispatchers. Only two of the real-life studies surveyed callers [6, 12] regarding their experience, and response rates were relatively low (below 22% in both studies), making it challenging to conclude what impact that video livestreaming may have on callers. Evidence from our review also suggests that video livestreaming could improve dispatchers’ clinical assessment and decision-making. Currently, dispatchers’ situational awareness and decision-making skills rely on the ability to obtain information about the incident and on their verbal communication skills [36]. Our review did not find any evidence of how or why video livestreaming impacts on decision-making processes, highlighting a gap in evidence.

Challenges included potential for additional dispatcher workload, stress, and the emotional impact of viewing video footage. In the simulation studies it was not possible to decipher whether the stress was partly induced by the simulated scenarios. Research has shown that simulation often creates anxiety and stress in participants [37]. Nevertheless, dispatchers are known to be at increased risk of stress, anxiety and depression [38]. New working practises, such a video livestreaming, need to consider approaches to minimise potential additional stress to dispatchers.

Our review raised important questions whether video livestreaming should be used selectively for specific conditions, high/low acuity, or for every call. As outlined in our results, most of the included studies investigated the use of video livestreaming for CPR only, reporting a range of positive outcomes [14–16, 23, 26], indicating usefulness in this area. In one of the real-life studies [6] the dispatchers chose when to initiate video livestreaming. Video was used more frequently with children and unconscious patients, described as “unable to talk for themselves”. Another study [12] investigated the use of video triage for low-acuity calls (such as diarrhoea, vomiting, and urine infection) during covid-19. Over half of the category 5 (low acuity) calls were upgraded after the video consultation, representing a potential important use for low-acuity calls. Further research is needed to understand for what type of calls video livestreaming is most beneficial.

The results of our review highlighted several additional significant topics that need to be considered when conducting research and implementing video livestreaming into practice. First, improving public awareness about the use of livestreaming by emergency services may help to increase caller acceptance and compliance rates, as highlighted in a study in South Korea, where dispatcher-assisted video CPR has been in use for some time [14]. Second, a number of the studies [13, 19] also emphasised the importance of training dispatchers in using video technology. Our results indicate that dispatchers may need training in developing new skills in using video livestreaming and to help them integrate video into existing workflows. Internationally, there are several types of emergency medical dispatch systems and the professional background of call takers and dispatchers varies from no healthcare training to being trained health professionals with extensive experience [4]. The impact of professional/clinical backgrounds on using livestreaming is currently unknown. Future implementation of video livestreaming in this context is likely to require bespoke training programmes that consider local and national medical emergency systems and processes. Indeed, Linderoth et al. [6] argued that dispatcher training to use video livestreaming needs a comprehensive approach as the additional visual information may add further complexity to the decision-making process. Third, our review also drew attention to the need to carefully review dispatch protocols and organisational systems to ensure that they are compatible with video livestreaming technology. Fourth, dispatchers in one Norwegian study [26] voiced concern about the potential misuse of the videos and the loss of identity protection. Two-way video communication (where caller and dispatcher could see each other) was used in seven of the studies, raising potential concerns regarding privacy and identity for dispatchers. Overall, our review found limited information regarding the ethical aspects of video livestreaming from medical emergency callers’ smartphones. Many of the studies had local research ethical review only, or ethical review approval ‘waived’ and provided limited information regarding the caller and/or patient consent processes. Two of the studies [13, 18] mentioned that dispatchers told callers during the consent process that the video was not being stored and callers’ smartphones could not be accessed. Future research needs to explore key ethical issues, such as privacy, dignity, information-governance and provide recommendations regarding how to best approach caller and patient consent.

Key gaps identified by our review were that few studies included data collected specifically from the callers and none of the studies included any health economic analysis. Only three of the studies included analysis of change in dispatch decisions regarding resources sent to scenes of incidents. There was also a lack of qualitative studies exploring the experiences of callers, dispatchers, and patients. Future research would benefit from including ethnographic observations ‘in the field’, taking account of important organisational factors and prevailing cultures, as these are known to have an impact on the implementation of new technologies [39], such as video livestreaming.

### Strengths and limitations

The review used a comprehensive search strategy that enabled capture and synthesis of international evidence related to the use of video livestreaming in this context. It uniquely synthesises the potential benefits, challenges and opportunities to inform future research and practice. The review included both real-life and simulation studies and presented the results separately which allowed a systematic overview of evidence base. The review was limited to papers written in the English Language and it is possible that it therefore omits some key international literature. The chosen search terms may have limited inclusion, although a broader search strategy was initially used, in order to determine how best to use limiters to only focus on relevant literature.

## Conclusion

The evidence base into the use of video livestreaming in this context is sparse. There are few studies of real-life practice, studies generally employ weak study designs, and caller perspectives are lacking. Over half of the studies focussed on dispatcher-assisted CPR. Nevertheless, there is growing evidence of potential opportunities to using this technology, including for improved clinical assessment and decision-making. These need to be balanced with consideration of the challenges uncovered in this review. Further well-designed robust research is needed, in particular into the experiences, acceptability and attitudes of both callers and dispatchers in real-life studies, and robust evidence of the effectiveness and cost effectiveness of implementing such technologies.

### Supplementary Information

Supplementary Material 1.

Supplementary Material 2.

## Data Availability

All data generated or analysed during this study are included in this published article.

## Abbreviations

AED: Automated External Defibrillators
CAD: Computer Aided Dispatch
CBD: Criteria-based dispatch
CPR: Cardiopulmonary resuscitation
EMDC: Emergency Medical Dispatch Centre
EMS: Emergency Medical Services
MESH: Medical Subject Headings
MPDS: Medical Priority Dispatch System
OHCA: Out-of-hospital cardiac arrest
ROSC: Return of Spontaneous Circulation
V-CPR: Video CPR
V-DACPR: Video-instructed dispatcher-assisted bystander cardiopulmonary resuscitation

## References

[1] Greenhalgh T, Wherton J, Shaw S, Morrison C (2020). Video consultations for covid-19. BMJ.

[2] Duke M, Tatum D, Sexton K, Stuke L, Robertson R, Sutherland M (2018). When minutes fly by: what is the true “golden hour” for air care?. Am Surg.

[3] Schellhaaß A, Popp E (2014). [Air rescue: current significance and practical issues]. Anaesthesist.

[4] Bohm K, Kurland L (2018). The accuracy of medical dispatch - a systematic review. Scand J Trauma Resusc Emerg Med.

[5] Eaton G, Brown S, Raitt J (2018). HEMS dispatch: a systematic review. Trauma.

[6] Linderoth G, Lippert F, Østergaard D, Ersbøll AK, Meyhoff CS, Folke F (2021). Live video from bystanders’ smartphones to medical dispatchers in real emergencies. BMC Emerg Med.

[7] Lin YY, Chiang WC, Hsieh MJ, Sun JT, Chang YC, Ma MHM (2018). Quality of audio-assisted versus video-assisted dispatcher-instructed bystander cardiopulmonary resuscitation: a systematic review and meta-analysis. Resuscitation.

[8] Bielski K, Böttiger BW, Pruc M, Gasecka A, Sieminski M, Jaguszewski MJ (2022). Outcomes of audio-instructed and video-instructed dispatcher-assisted cardiopulmonary resuscitation: a systematic review and meta-analysis. Ann Med.

[9] Sýkora R, Peřan D, Renza M, Bradna J, Smetana J, Duška F (2022). Video emergency calls in medical dispatching: a scoping review. Prehosp Disaster Med.

[10] Arksey H, O’Malley L (2005). Scoping studies: towards a methodological framework. Int J Soc Res Methodol.

[11] Tricco AC, Lillie E, Zarin W, O’Brien KK, Colquhoun H, Levac D (2018). PRISMA extension for scoping reviews (PRISMA-ScR): checklist and explanation. Ann Intern Med.

[12] Bell F, Pilbery R, Connell R, Fletcher D, Leatherland T, Cottrell L (2021). The acceptability and safety of video triage for ambulance service patients and clinicians during the COVID-19 pandemic. Br Paramedic J.

[13] Idland S, Iversen E, Brattebø G, Kramer-Johansen J, Hjortdahl M (2022). From hearing to seeing: medical dispatchers’ experience with use of video streaming in medical emergency calls – a qualitative study. BMJ Open.

[14] Lee HS, You K, Jeon JP, Kim C, Kim S (2021). The effect of video-instructed versus audio-instructed dispatcher-assisted cardiopulmonary resuscitation on patient outcomes following out of hospital cardiac arrest in Seoul. Sci Rep.

[15] Lee SY, Song KJ, Shin SD, Hong KJ, Kim TH (2020). Comparison of the effects of audio-instructed and video-instructed dispatcher-assisted cardiopulmonary resuscitation on resuscitation outcomes after out-of-hospital cardiac arrest. Resuscitation.

[16] Linderoth G, Rosenkrantz O, Lippert F, Østergaard D, Ersbøll AK, Meyhoff CS (2021). Live video from bystanders’ smartphones to improve cardiopulmonary resuscitation. Resuscitation.

[17] Neustaedter C, Jones B, O’Hara K, Sellen A (2018). The benefits and challenges of video calling for emergency situations. Proceedings of the 2018 CHI Conference on Human Factors in Computing Systems.

[18] Steen-Tveit K, Munkvold BE, Radianti J (2021). Using live video for communication between lay bystanders and emergency dispatchers in command and control centres. Int J Emerg Manag.

[19] Ter Avest E, Lambert E, de Coverly R, Tucker H, Griggs J, Wilson MH (2019). Live video footage from scene to aid helicopter emergency medical service dispatch: a feasibility study. Scand J Trauma Resusc Emerg Med.

[20] Bang JY, Cho Y, Cho GC, Lee J, Kim IY (2020). Can mobile videocall assist laypersons’ use of automated external defibrillators? A randomized simulation study and qualitative analysis. Biomed Res Int.

[21] Bolle SR, Johnsen E, Gilbert M (2011). Video calls for dispatcher-assisted cardiopulmonary resuscitation can improve the confidence of lay rescuers–surveys after simulated cardiac arrest. J Telemed Telecare.

[22] Bolle SR, Scholl J, Gilbert M (2009). Can video mobile phones improve CPR quality when used for dispatcher assistance during simulated cardiac arrest?. Acta Anaesthesiol Scand.

[23] Ecker H, Wingen S, Hagemeier A, Plata C, Bottiger B, Wetsch W (2022). Dispatcher self-assessment and attitude toward video assistance as a new tool in simulated cardiopulmonary resuscitation. WestJEM.

[24] Ecker H, Wingen S, Hamacher S, Lindacher F, Böttiger BW, Wetsch WA (2021). Evaluation of CPR quality via smartphone with a video livestream - a study in a metropolitan area. Prehosp Emerg Care.

[25] Igarashi Y, Suzuki K, Norii T, Motomura T, Yoshino Y, Kitagoya Y (2022). Do video calls improve dispatcher-assisted first aid for infants with foreign body airway obstruction? A randomized controlled trial/simulation study. J Nippon Med Sch.

[26] Johnsen E, Bolle SR (2008). To see or not to see—better dispatcher-assisted CPR with video-calls? A qualitative study based on simulated trials. Resuscitation.

[27] Kim HJ, Kim JH, Park D (2021). Comparing audio- and video-delivered instructions in dispatcher-assisted cardiopulmonary resuscitation with drone-delivered automatic external defibrillator: a mixed methods simulation study. PeerJ.

[28] Lee SGW, Kim TH, Lee HS, Shin SD, Song KJ, Hong KJ (2021). Efficacy of a new dispatcher-assisted cardiopulmonary resuscitation protocol with audio call-to-video call transition. Am J Emerg Med.

[29] Melbye S, Hotvedt M, Bolle SR (2014). Mobile videoconferencing for enhanced emergency medical communication - a shot in the dark or a walk in the park? –– A simulation study. Scand J Trauma Resusc Emerg Med.

[30] Perry O, Wacht O, Jaffe E, Sinuany-Stern Z, Bitan Y (2020). Using a filming protocol to improve video-instructed cardiopulmonary resuscitation. Technol Health Care.

[31] Peters M, Stipulante S, Cloes V, Mulder A, Lebrun F, Donneau AF (2022). Can video assistance improve the quality of pediatric dispatcher-assisted cardiopulmonary resuscitation?. Pediatr Emerg Care.

[32] Stipulante S, Delfosse AS, Donneau AF, Hartsein G, Haus S, D’Orio V (2016). Interactive videoconferencing versus audio telephone calls for dispatcher-assisted cardiopulmonary resuscitation using the ALERT algorithm: a randomized trial. Eur J Emerg Med.

[33] Yang C-W, Wang H-C, Chiang W-C, Chang W-T, Yen Z-S, Chen S-Y (2008). Impact of adding video communication to dispatch instructions on the quality of rescue breathing in simulated cardiac arrests–a randomized controlled study. Resuscitation.

[34] You JS, Park S, Chung SP, Park JW (2008). Performance of cellular phones with video telephony in the use of automated external defibrillators by untrained laypersons. Emerg Med J.

[35] Hall A, Wooton K, Hutton A. Bystander Experiences at and after a Motor Vehicle Accident: A review of the literature. Australas J Paramed. 2013;10:1–10.

[36] Møller TP, Jensen HG, Viereck S, Lippert F, Østergaaard D (2021). Medical dispatchers’ perception of the interaction with the caller during emergency calls - a qualitative study. Scand J Trauma Resusc Emerg Med.

[37] Paskins Z, Peile E (2010). Final year medical students’ views on simulation-based teaching: a comparison with the best evidence medical education systematic review. Med Teach.

[38] Smith EC, Holmes L, Burkle FM (2019). Exploring the physical and mental health challenges associated with emergency service call-taking and dispatching: a review of the literature. Prehosp Disaster Med.

[39] Jennett P, Yeo M, Pauls M, Graham J (2003). Organizational readiness for telemedicine: implications for success and failure. J Telemed Telecare.

