# Supplementary material for: Video livestreaming from medical emergency callers’ smartphones to emergency medical dispatch centres: a scoping review of current uses, opportunities, and challenges
Source: BMC Emerg Med. 2024 Jun 11;24:99. doi: 10.1186/s12873-024-01015-9 (PMC11165798; doi:10.1186/s12873-024-01015-9)
Supplement: Supplementary file 2 — Supplementary Material 2. [file 12873_2024_1015_MOESM2_ESM.docx]

Supplementary File 2: MEDLINE search strategy

| **#** | **Searches** |
| --- | --- |
| 1-10 | AB "pre*hospital" OR TI "pre*hospital" AB "out of hospital" OR TI "out of hospital"  AB "emergency medical service*" OR TI "emergency medical service*  AB "ambulance service*" OR TI "ambulance service*"  AB dispatch* OR TI dispatch*  AB "call*taker*" OR TI "call*taker*  (MH "Air Ambulances")  (MH "Emergency Medical Services")  (MH "Emergency Medical Dispatcher")  (MH "Emergency Medical Dispatch")  (MH "Emergency Medical Service Communication Systems") |
| 11 | S1 OR S2 OR S3 OR S4 OR S5 OR S6 OR S7 OR S8 OR S9 OR S10 |
| 12-30 | (MH "Cell Phone Use")  (MH "Cell Phone")  (MH "Smartphone") OR (MH "Mobile Applications")  AB "video* transmission*" OR TI "video* transmission*"  (MH "Audiovisual Aids")  (MH "Videoconferencing")  (MH "Telemedicine")  (MH "Video Recording")  AB "video* based" OR TI "video* based"  AB "mobile health technology*" OR TI "mobile health technology*"  AB "video* conferencing" OR TI "video* conferencing"  AB "video* communication*" OR TI "video* communication*"  AB "tele*medicine" OR TI "tele*medicine"  AB "visual information" OR TI "visual information"  AB "video*call*" OR TI "video*call*"  AB "mobile video*" OR TI "mobile video*"  AB "live video footage*" OR IT "live video footage*  AB "live*stream*" OR TI "live*stream*"  AB video* OR TI video* |
| 31 | S12 OR S13 OR S14 OR S15 OR S16 OR S17 OR S18 OR S19 OR S20 OR S21 OR S22 OR S23 OR S24 OR S25 OR S26 OR S27 OR S28 OR S29 OR S30 |
| 32 | **S11 AND S31 (limited to yr=2007-current)** |
